# MicroRNA-613 represses lipogenesis in HepG2 cells by downregulating LXRα

**DOI:** 10.1186/1476-511X-12-32

**Published:** 2013-03-08

**Authors:** Dan Zhong, Yan Zhang, Yi-jun Zeng, Min Gao, Geng-ze Wu, Chang-jiang Hu, Gang Huang, Feng-tian He

**Affiliations:** 1 Department of Biochemistry and Molecular Biology, College of Basic Medical Sciences, Third Military Medical University, Chongqing 400038, China; 2 Department of cardiology, Daping Hospital, Third Military Medical University, Chongqing 400042, China

**Keywords:** microRNA-613, Lipogenesis, Liver X receptor α, HepG2 cells

## Abstract

**Background:**

MicroRNAs (miRNAs) emerge as new important regulators of lipid homeostasis by regulating corresponding genes. MiR-613 is a newly discovered microRNA, of which the biological function is unknown. A recent report has shown that miR-613 downregulates liver X receptor α (LXRα), a ligand-activated nuclear receptor playing an important role in the regulation of lipid metabolism. The purpose of this study is to explore the effect and the molecular basis of miR-613 on lipogenesis in HepG2 cells.

**Methods:**

HepG2 cells were transiently transfected with miR-613 mimic or control microRNA. Real time PCR, Western blot, Luciferase reporter assay and Oil Red O staining were employed to examine the expression of LXRα and its target genes involved in lipogenesis, binding site for miR-613 in 3^′^-untranslated region (3^′^-UTR) of LXRα mRNA and lipid droplet accumulation in the cells.

**Results:**

MiR-613 dramatically suppressed the expression of LXRα and its target genes including sterol-regulatory element binding protein 1c (SREBP-1c), fatty acid synthase (FAS), carbohydrate responsive element-binding protein (ChREBP) and acetyl-CoA carboxylase (ACC). Reporter assay showed that miR-613 directly bound to 3^′^-UTR of LXRα mRNA. Moreover, miR-613 significantly repressed LXRα-induced lipid droplet accumulation in HepG2 cells. Ectopic expression of LXRα without 3^′^-UTR markedly attenuated the miR-613-mediated downregulation of LXRα’s target genes and LXRα-induced lipid droplet accumulation.

**Conclusions:**

MiR-613 suppresses lipogenesis by directly targeting LXRα in HepG2 cells, suggesting that miR-613 may serve as a novel target for regulating lipid homeostasis.

## Introduction

MicroRNAs (miRNAs) are a class of 22 nucleotide non-coding RNAs that regulate genes by binding to the 3^′^-untranslated region (3^′^-UTR) of target mRNAs. They are implicated in a variety of diseases including obesity, cancer, atherosclerosis and diabetes [1-3]. Recent studies have shown that miRNAs play an important role in lipid metabolism and several miRNAs have been identified, such as miR-33, miR-122, miR-27, miR-370 [4,5]. MiR-33 regulates the ATP-binding cassette transporters (ABC transporters), ABCA1 and ABCG1, in addition to its role in fatty acid β-oxidation. MiR-122 regulates several genes that control fatty acid (FA) and Triglyceride (TG) biosynthesis, such as fatty acid synthase (FAS), acetyl-CoA carboxylase 1 (ACC-1), acetyl-CoA carboxylase 2 (ACC-2), and sterol regulatory element-binding protein 1c (SREBP-1c), as well as genes that regulate fatty acid β-oxidation, such as carnitine palmitoyltransferase 1α (CPT1α) [6]. Similarly, other miRNAs regulate lipid homeostasis by targeting a variety of lipid metabolism–associated genes.

MiR-613 is a newly discovered miRNA, whose function and molecular basis in the biological process and diseases is incompletely understood. It is reported that miR-613 plays inhibitory role in repressing the Wnt pathway, but its effect on the Wnt-dependent biological processes is unclear. Another report demonstrates the negative role of miR-613 in a feedback loop in the auto-regulation of the human LXRα [7,8] without showing its subsequent function. Therefore, it is important to clarify the biological function of miR-613 in future.

Lipid homeostasis is regulated by a family of transcription factors including the nuclear hormone receptor LXRα [9]. The molecular mechanism responsible for LXRα-mediated lipogenesis has been largely attributed to the dramatic upregulation of the lipogenic genes such as SREBP-1c, FAS, ChREBP and ACC [10-14]. LXRα-induced activation of those genes can result in the enhancement of fatty acid synthesis, which contributes to a series of lipogenesis-associated diseases. Therefore, since miR-613 directly targets and decreases the expression of LXRα, further studies are needed to determine whether miR-613 can relief LXRα-induced lipogenesis via repression of LXRα expression.

In this study, we confirmed that miR-613 directly downregulated LXRα expression at both mRNA and protein levels. Subsequently, LXRα-induced lipogenic genes, such as SREBP-1c, FAS, ChREBP and ACC, were inhibited by miR-613, which was abolished by the ectopic expression of LXRα without 3^′^-UTR. Oil Red O staining revealed that miR-613 reduced LXRα-induced lipid droplet accumulation in HepG2 cells, which was also attenuated by ectopic expression of LXRα. Our study suggests miR-613 as a critical regulator of lipogenesis.

## Materials and methods

### Reagents

The miR-613 mimic (5^′^- AGGAAUGUUCCUUCUUUGCC -3^′^) and negative control (NC, 5^′^- UUCUCCGAACGUGUCACGUTT -3^′^) were synthesized by Shanghai GenePharma (Shanghai, China). TO901317 and GW3965 were purchased from Sigma Chemical Company (St Louis, MO, USA)

### Cell culture

Human hepatocellular carcinoma cell line HepG2 was purchased from ATCC and cultured in Dulbeco’s Modified Eagle’s medium (Gibco, Shanghai, China) supplemented with 10% fetal bovine serum (FBS) (Gibco, Gaithersburg, USA), streptomycin (100 μg/ml) and antibiotics (100 U/ml penicillin and 100 μg/ml streptomycin) at 37°C in 5% CO_2_ humid incubator.

### Plasmid construction

The DNA fragment corresponding to 76-113 nt of human LXRα 3^′^-UTR containing the miR-613 binding site was synthesized by Sangon Biotech and cloned into pMIR-REPORT vector (Invitrogen) at *Sac* I and *Hin*d III site downstream of the luciferase gene, and the resulting plasmid was named as pMIR/LXRαMIRE. Similarly, the DNA fragment containing mutations in miR-613 binding site was synthesized and cloned into pMIR-REPORT, and the resulting plasmid was named as pMIR/LXRαMIRE–mut. The 3^′^-UTR of the human LXRβ gene was amplified by PCR with the cDNA of HepG2 cells as template. Purified PCR products digested by *Sac* I and *Hin*d III (Takara) were cloned into pMIR-REPORT vector, and the resulting plasmid was named as pMIR-LXRβ.

### Transient transfections and luciferase assays

HepG2 cells, grown to 70% to 80% confluence, were transiently transfected with luciferase reporters (pMIR-REPORT, pMIR-LXRβ, pMIR/LXRαMIRE, pMIR/LXRαMIRE–mut) and miR-613 mimic or mimic NC, using Lipofectamine 2000 according to the manufacturer’s instructions (Invitrogen). Transfection efficiency was monitored by cotransfection of pMIR-REPORT-β-gal (Promega). Luciferase activities were measured using a luciferase assay system (Promega). Transfection experiments were performed 3 times in triplicate. Data was represented as fold induction over reporter gene treated with vehicle alone.

### Western blot

24 hours after transfection, cells were exposed to TO901317 (5 μM) for 24 hours. Dimethyl sulfoxide (DMSO, Sigma) was used as controls. Then the whole cell proteins were harvested in lysis buffer (50 mM Tris-base, 150 mM NaCl, 1.0 mM EDTA, 0.1% SDS, 1% sodium deoxycholate and 1% TritonX-100) containing protease inhibitor cocktail. And the protein concentrations were determined using Bradford protein assay reagent (Bio-Rad). Subsequently, the total proteins (40 μg/well) were separated with 10% SDS-PAGE and transferred to PVDF (polyvinylidene fluoride) membranes (Millipore). After blocked 1 hour with 5% nonfat milk, the membranes were incubated with mouse anti-human LXRα antibody (Abcam), or mouse anti-human β-actin antibody (Santa Cruz Biotechnology) at 4°C over night. After washing with TBST, blots were incubated with HRP-labeled rabbit anti-mouse IgG (Invitrogen) for 1 hour at room temperature. Detection was achieved using enhanced chemiluminescence reagents (Pierce) and exposure to film.

### Real-time quantitative reverse transcription-polymerase chain reaction (qRT-PCR)

For qRT-PCR, total RNA was extracted from the HepG2 cells using TRIzol and the first-strand cDNA was synthesized using M-MLV reverse transcriptase (Invitrogen) and oligo (dT) primer according to the manufacturer’s manual. The expression of LXRα, SREBP-1c, FAS, ChREBP and ACC mRNA was examined by qPCR using SYBR green-based assays. Relative expressions were calculated with normalization to β-actin values by using the 2^-△△Ct^ method. The sequences of primers used for quantitative PCR analysis were shown in Table 1.

**Table 1:** Primers used for PCR

**Primer name**	**Sequence**
LXRα 3^′^-UTR Forward	5^′^-CTGTTCTGTCCCCATATTTTCTG-3^′^
LXRα 3^′^-UTR Reverse	5^′^-TCGCAACCCTTTGACTCTCT-3^′^
LXRβ 3^′^-UTR Forward	5^′^-GACCACCCTCCAGCAGATAG-3^′^
LXRβ 3^′^-UTR Reverse	5^′^-AGAGGAAGGCCCTGGTCTC-3^′^
LXRα Forward	5^′^-TCAGAGAGGAAGCCAGGATG-3^′^
LXRα Reverse	5^′^-ACGGATCTCTGTGGGTTCTG-3^′^
SREBP-1c Forward	5^′^-CGACATCGAAGACATGCTTCAG-3^′^
SREBP-1c Reverse	5^′^-GGAAGGCTTCAAGAGAGGAGC-3^′^
FAS Forward	5^′^-GACATCGTCCATTCGTTTGTG-3^′^
FAS Reverse	5^′^-CGGATCACCTTCTTGAGCTCC-3^′^
ChREBP Forward	5^′^-AGAGACAAGATCCGCCTGAA-3^′^
ChREBP Reverse	5^′^-CTTCCAGTAGTTCCCTCCA-3^′^
ACC Forward	5^′^-GCTGCTCGGATCACTAGTGAA-3^′^
ACC Reverse	5^′^-TTCTGCTATCAGTCTGTCCAG-3^′^
β-actin Forward	5^′^-GTGAAGGTGACAGCAGTCGGTT-3^′^
β-actin Reverse	5^′^-GAAGTGGGGTGGCTTTTAGGA-3^′^

### Oil Red O staining

Oil Red O stock solution was prepared in isopropanol (0.25 g/100 ml) and heated to 100°C for 10 min. TO901317-treated cells were fixed with 4% paraformaldehyde for 30 min and washed with PBS. Then cells were soaked in 60% Oil Red O stock solution diluted by distilled water for 30 min. Stained cells were washed with PBS until the background became clear. Images were captured with fluorescence microscopy (Olympus).

### Statistical analysis

All data are expressed as means ± SD unless otherwise stated. Comparisons between two groups were made with unpaired Student’s t-tests. Non-parametric comparisons between three or more groups were made with ANOVA followed by Kruskal–Wallis post hoc analysis. In all cases, *P* < 0.05 was considered statistically significant.

## Results

### MiR-613 reduces LXRα expression at both mRNA and protein levels

**Figure 1:**
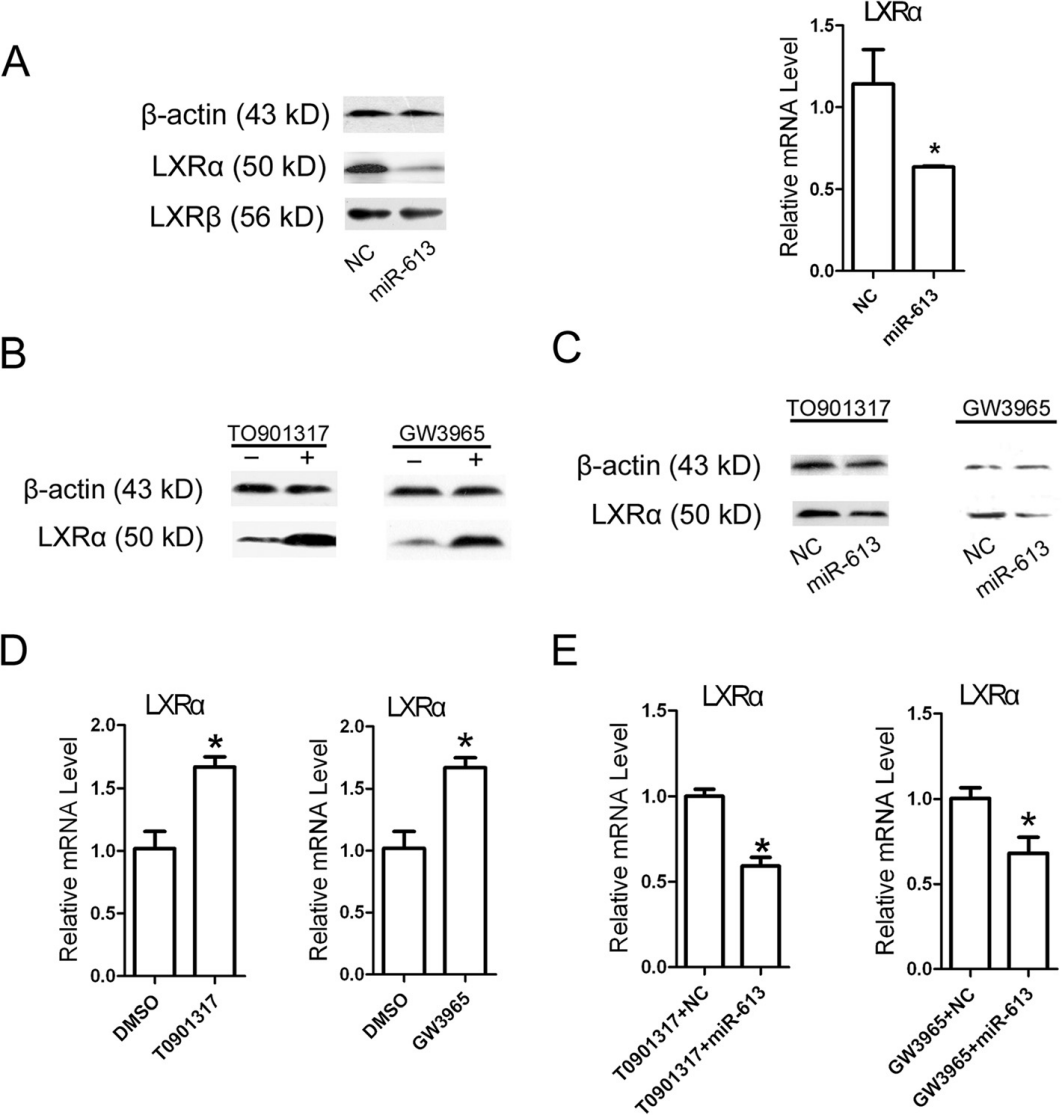
**MiR-613 decreases LXRα expression at both mRNA and protein levels. A**: HepG2 cells were transfected with 80 nM miR-613 mimic or mimic negative control (NC), after 24 hours total protein was subjected to Western blotting analysis and mRNA expression levels of LXRα were analyzed by real-time quantitative PCR and normalized with β-actin control. **B** and **D**, HepG2 cells were treated with TO901317 (5 μM) or GW3965 (2 μM) for 24 hours. Western blot analysis for LXRα protein level (**B**) and Real-time PCR analysis for LXRα mRNA level (**D**). **C** and **E**, 12 hours after transfected with 80 nM miR-613 mimic or NC, HepG2 cells were treated with TO901317 (5 μM) or GW3965 (2 μM) for 24 hours. Western blot analysis for LXRα protein level (**C**) and Real-time PCR analysis for LXRα mRNA level (**E**). The relative level of LXRα expression determined using the 2-^△△CT^ method. *, *P* < 0.05 (n = 3 for each group).

It has been reported that miR-613 negatively regulated the expression and activity of endogenous LXRα. To examine whether these results are obtainable under our experimental conditions, HepG2 cells were transfected with miR-613 mimic or negative control (NC). Western blot and real-time PCR analysis showed that endogenous LXRα expression was repressed by miR-613 at both protein and mRNA levels (Figure 1A), while LXRβ expression was not affected, which suggested that miR-613 effect was specific for LXRα. Furthermore, as LXRα activation leads to the lipogenesis in liver, we determined whether miR-613 could inhibit agonist-induced LXRα expression. Treatment with either TO901317 or GW3965 induced mRNA and protein expression of LXRα (Figure 1B and D), which was repressed by miR-613 (Figure 1C and E). Similarly, miR-613 suppressed TO901317-induced LXRα expression at both mRNA and protein levels in L02 cells (Additional file 1: Figure S1). These results demonstrated that miR-613 downregulated both endogenous and agonist-induced LXRα expression.

### MiR-613 directly targets LXRα 3^′^-UTR

**Figure 2:**
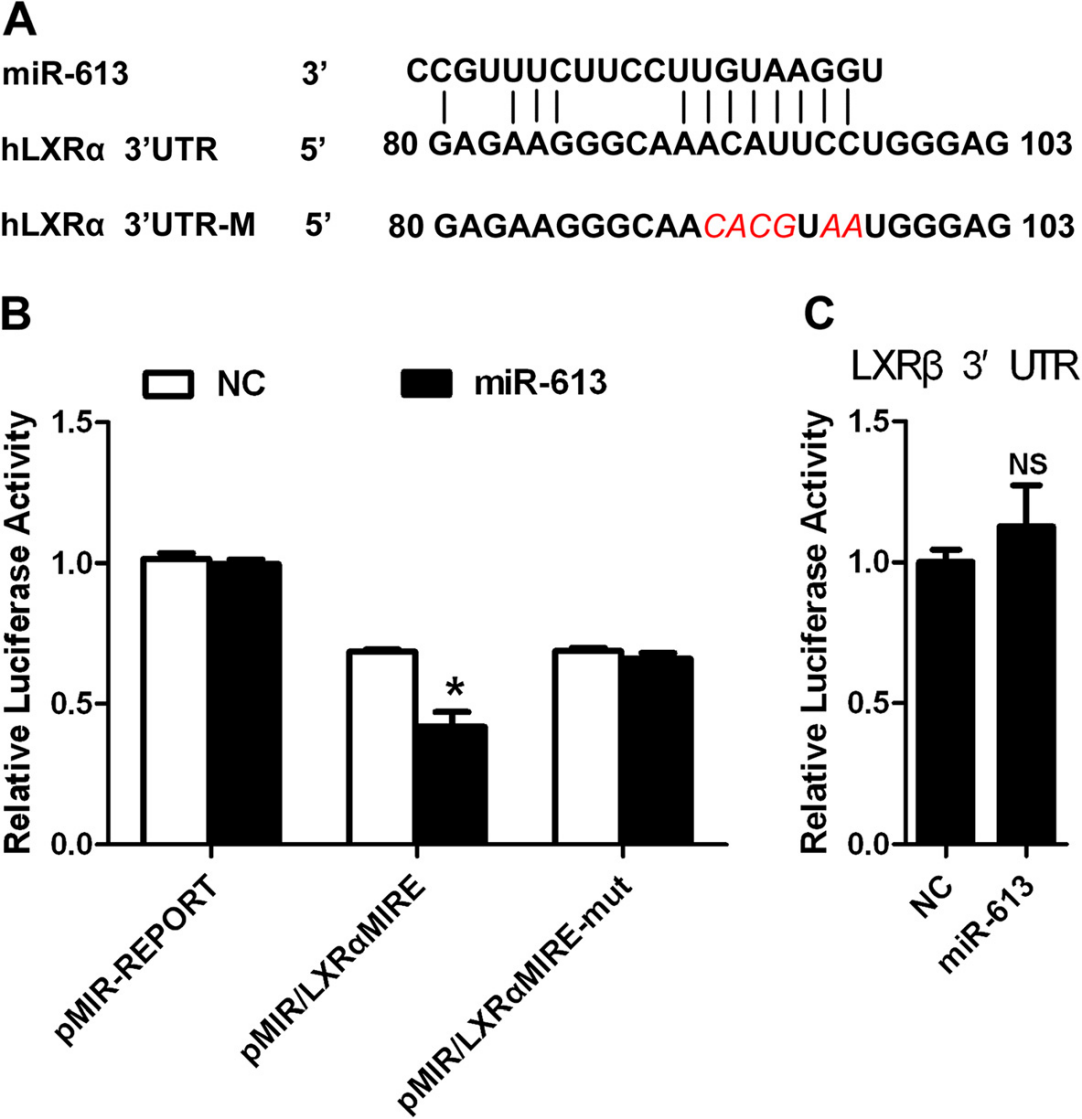
**MiR-613 directly targets LXRα 3**^′^**-UTR. A**: A putative binding site of miR-613 in the 3^′^-UTR of LXRα mRNA and mutated nucleotide residues are shown in red and *italics*. **B**: pMIR/LXRαMIRE or pMIR/LXRαMIRE-mut was cotransfected with 80 nM miR-613 mimic or NC into HepG2 cells. **C**, pMIR-LXRβ was transiently cotransfected with 80 nM indicated RNA oligonucleotides into HepG2 cells. Luciferase assay was conducted after 24 hours. The data was the firefly luciferase activities normalized with the β-galactosidase activities. *, *P* < 0.05 (n = 3 for each group).

We also found the binding site for miR-613 on LXRα 3^′^-UTR by TargetScan analyses (Figure 2A). Cotransfection with miR-613 mimic resulted in a decrease of the luciferase activity of pMIR/LXRαMIRE plasmid containing the LXRαMIRE613, while the luciferase activity of pMIR/LXRαMIRE-mut plasmid containing mutations in miR-613 recognition site was not affected (Figure 2B). Moreover, cotransfection with miR-613 mimic had no effect on the luciferase activity of pMIR-LXRβ plasmid containing the LXRβ 3^′^-UTR (Figure 2C). These results revealed that miR-613 directly targeted LXRα on its 3^′^-UTR, but not LXRβ.

### MiR-613 suppresses lipogenic genes through downregulation of LXRα

**Figure 3:**
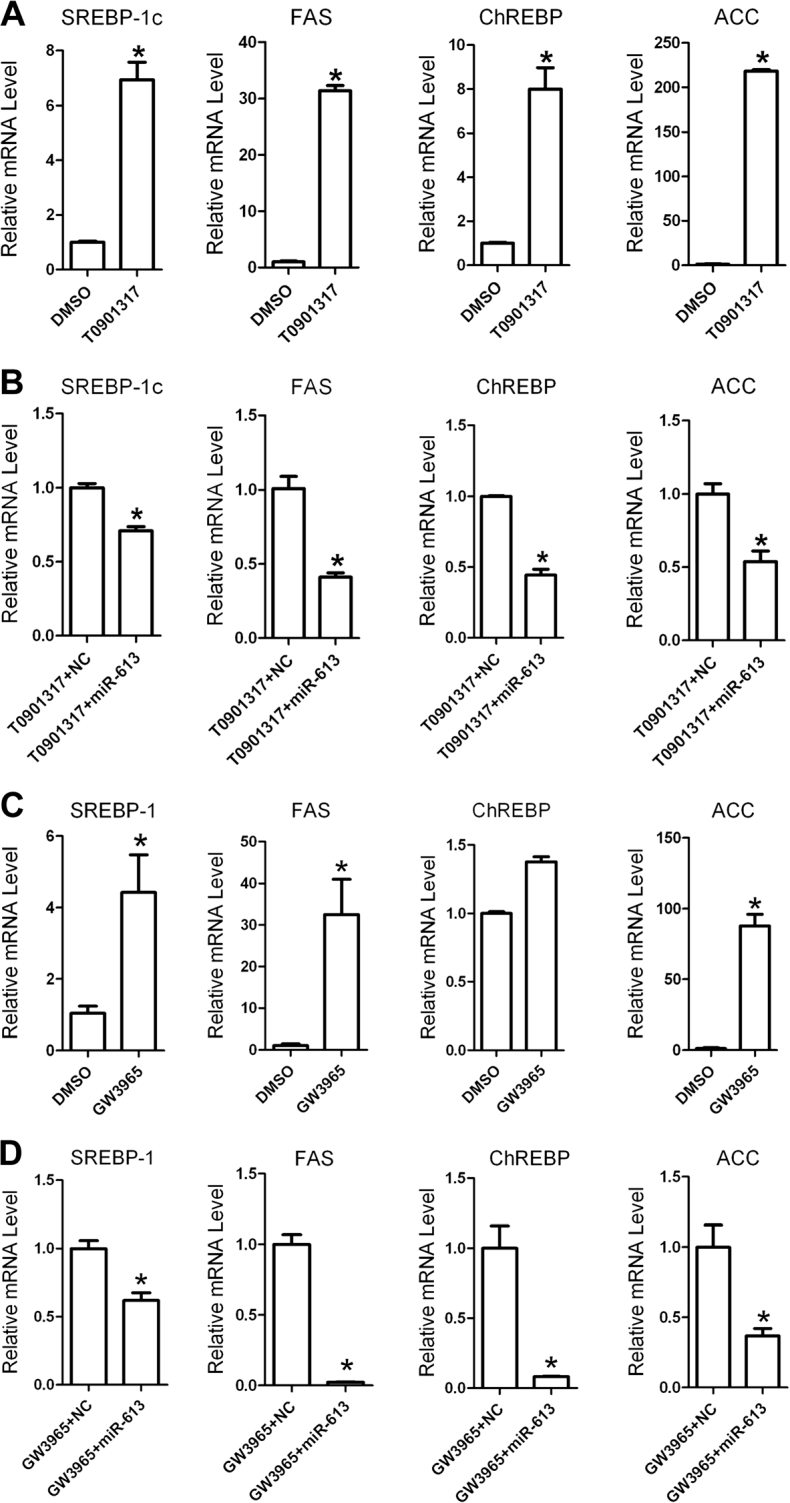
**MiR-613 suppresses LXRα-induced lipogenic genes. A** and **B**: HepG2 cells were treated with TO901317 (5 μM) or GW3965 (2 μM) for 24 hours. Real-time PCR analysis for SREBP-1c, FAS, ChREBP and ACC mRNA level. **C** and **D**, after 12 hours transfected with 80 nM miR-613 mimic or NC, HepG2 cells were treated with TO901317 (5 μM) or GW3965 (2 μM) for 24 hours. Real-time PCR analysis for SREBP-1c, FAS, ChREBP and ACC mRNA level. The relative level of lipogenic gene expression determined using the 2-^△△CT^ method. *, *P* < 0.05 (n = 3 for each group).

**Figure 4:**
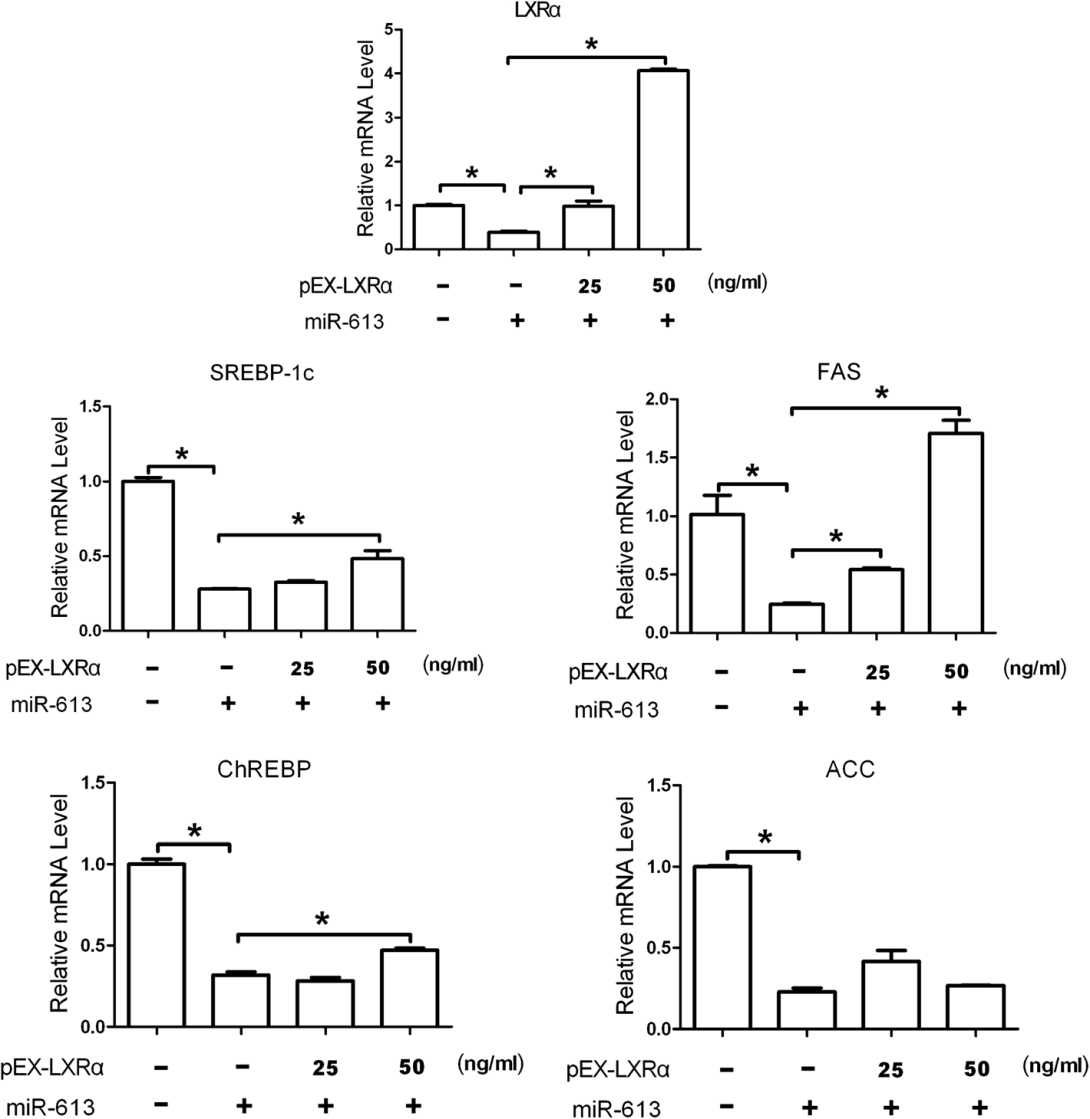
**MiR-613 represses lipogenic genes by mediating LXRα.** HepG2 cells were cotransfected with 80 nM miR-613 mimic or NC and LXRα-expressing plasmid pEX-LXRα (without 3^′^-UTR in LXRα mRNA) for 24 hours. The SREBP-1c, FAS, ChREBP and ACC expression was normalized to β-actin mRNA expression, respectively. The relative level of indicated gene expression determined using the 2-^△△CT^ method. *, *P*<0.05 (n=3 for each group).

Activation of LXRα has been shown to promote the hepatic lipogenesis by increasing the expression of lipogenic genes. We therefore determined the effect of miR-613 on LXRα-induced genes related with lipogenesis. As is shown in Figure 3A and C, both TO901317 and GW3965 successfully upregulated the mRNA expression of LXRα-targeted lipogenic genes, such as SREBP-1c, FAS, ChREBP and ACC. Moreover, miR-613 significantly decreased LXRα-induced expression of those genes (Figure 3B and D). Furthermore, miR-613 also downregulated these TO901317-induced target genes in L02 cells (Additional file 2: Figure S2). To examine whether miR-613 repressed lipogenic genes via downregulation of LXRα, HepG2 cells were cotransfected with miR-613 mimic and LXRα-expressing plasmid pEX-LXRα (without 3^′^-UTR in LXRα mRNA). As expected, ectopic expression of LXRα reversed the inhibitory effect of miR-613 on lipogenic genes (Figure 4). These results suggested that miR-613 repressed lipogenic genes in an LXRα-dependent manner.

### MiR-613 reduces lipid droplet accumulation through downregulation of LXRα in HepG2 cells

**Figure 5:**
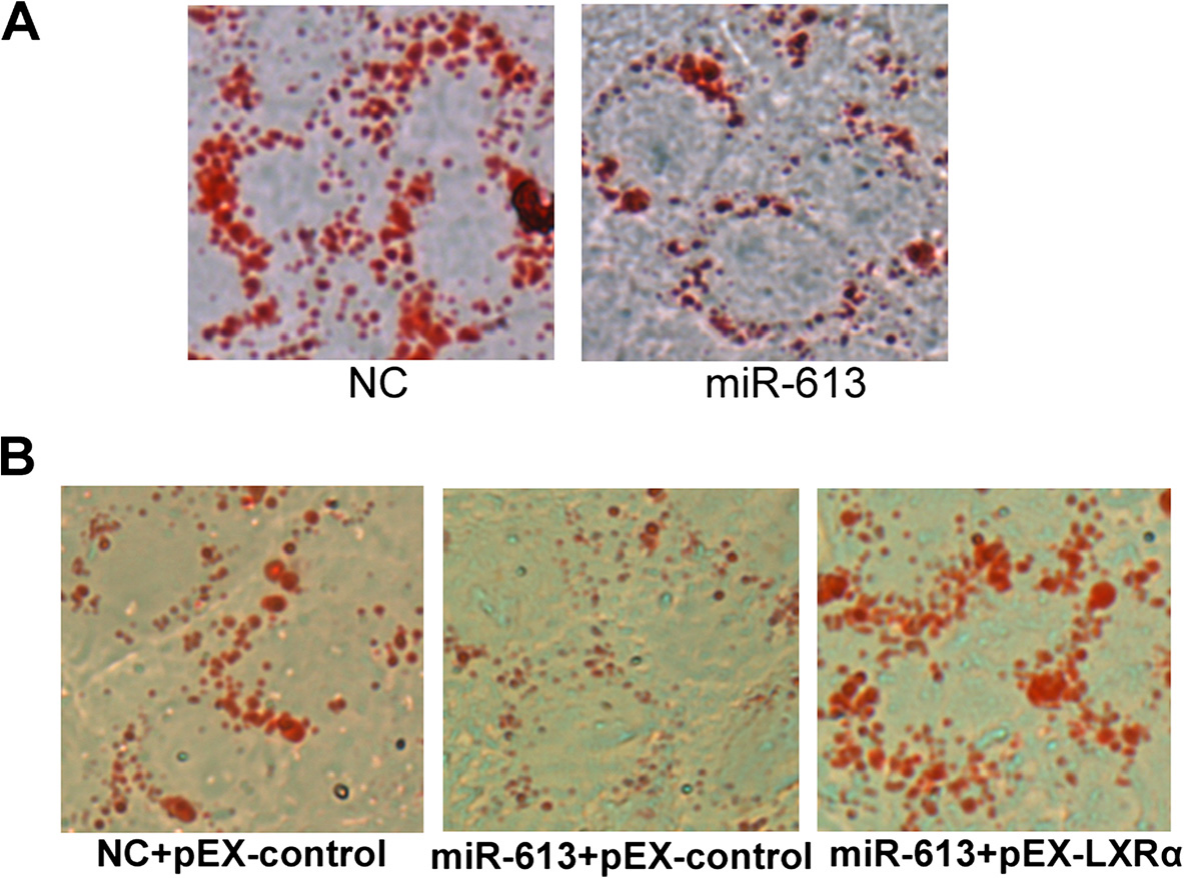
**MiR-613 represses lipogenesis by mediating LXRα.** The lipid synthesis was shown by Oil Red O staining (400×). **A**: After 12 hours transfected with 80 nM miR-613 mimic or NC, HepG2 cells were treated with TO901317 (5 μM) or GW3965 (2 μM) for 24 hours. **B**: HepG2 cells were cotransfected with 80 nM miR-613 mimic or NC and LXRα-expressing plasmid pEX-LXRα for 24 hours. Results represent 3 independent experiments.

To further investigate the function of miR-613, we determined whether miR-613 could decrease LXRα-induced accumulation of lipid droplets in HepG2 cells. 12 hours after transfected with miR-613 mimic, HepG2 cells were treated with TO901317 for 24 hours, then Oil Red O staining was performed. The lipid droplet accumulation of cells transfected with miR-613 mimic was markedly reduced compared with negative controls (Figure 5A). However, ectopic expression of LXRα (without 3^′^-UTR) attenuated the suppression of lipid droplet accumulation by miR-613 (Figure 5B). These results suggested that miR-613 might function as an anti-lipogenesis miRNA in HepG2 cells.

## Discussion

With the development of our modern society, the people’s lifestyle is characterized by an overabundant supply of food rich in energy and little physical activity, which enhance the lipid accumulation leading to a series of lipogenesis-associated diseases including dyslipidemia, hypertension, heart disease and fatty liver [15-18]. Therefore, the negative regulation of lipid synthesis may help with the treatment for lipid metabolism disorders. In our present study, we found miR-613 as an anti-lipogenesis miRNA through the suppression of LXRα, which provided a novel effective target for the therapy of lipogenesis-associated diseases.

From the recent studies, our understanding of miR-613 is extremely limited. It is reported that miR-613 is involved in the auto-regulation of LXRα by directly targeting LXRα 3^′^-UTR, but its biological function isn’t mentioned. Here, we first revealed the anti-lipogenic function of miR-613. It repressed expression of lipogenic genes and accumulation of lipid droplets in HepG2 cells by suppression of LXRα, which suggested the potential use of miR-613 as a therapeutic tool in lipogenesis-associated diseases, such as hepatic steatosis. Another report has demonstrated that miR-613 has inhibitory effect on the activity of the Wnt pathway by targeting the pathway upstream of Axin or active β-catenin. However, there is no evidence showing the effect of miR-613 on Wnt-dependent human cancer cells and the precise and direct targets of miR-613 [7,8]. Consequently, both the effects of miR-613 on Wnt-dependent cell proliferation and its other biological functions remain to be clarified. Moreover, for one miRNA can target more than one gene, further studies are needed to establish target landscape of miR-613.

MiRNAs regulate gene expression by binding mRNAs at 3^′^-UTR/5^′^-UTR or open reading frame (ORF), leading to translational repression and mRNA cleavage [19-21]. Though the targeting site for miR-613 at LXRα 3^′^-UTR has been uncovered, further study is needed to find out whether miR-613 can regulate LXRα by binding at 5^′^-UTR or ORF of LXRα mRNA. Unfortunately, bioinformatic analysis showed no possible binding site for miR-613 at ORF of LXRα mRNA, which might not be the mechanism by which miR-613 regulates LXRα.

Increasing genes are shown to be in the control of LXRα, but the regulation of LXRα itself is not fully understood. It has been reported that peroxisome proliferator-activated receptor (PPAR), retinoid-related orphan receptor (ROR), protein kinase C (PKC) and oxysterol binding protein related protein 1S (ORP1S) can potentially regulate the activation of LXRα [22-25]. In addition, the epigenic modification such as phosphorylation and SUMOylation, may affect LXRα actions [26,27]. Thus there are opportunities for miR-613 to indirectly regulate LXRα through the modulation of its upstream regulators such as PPAR, ROR and PKC. Similarly, although we demonstrated that miR-613 indirectly affected the expression of lipogenic genes, it needs to be examined whether the miRNA could directly target these lipogenic genes.

## Conclusions

In our present study, we demonstrated that miR-613 function as a novel modulater participating in lipid metabolism. It repressed lipogenic genes including SREBP-1c, FAS, ChREBP and ACC by suppression of LXRα expression. Subsequently, the accumulation of lipid droplets in HpG2 cells was significantly decreased by miR-613. These findings suggested that miR-613 was a potential target for the therapy of lipid dysfunction associated diseases.

## Abbreviations

LXRα/β: Liver X receptor α/β; miRs: MicroRNAs; SREBP-1c: Sterol-regulatory element binding protein 1c; FAS: Fatty acid synthase; ChREBP: Carbohydrate responsive element-binding protein; ACC: Acetyl-CoA carboxylase; RXRα: Retinoid X receptor α; PPAR: Peroxisome proliferator-activated receptor; ROR: Retinoid-related orphan receptor; PKC: Protein kinase C; ORP1S: Oxysterol binding protein related protein 1S; CPT1α: Carnitine palmitoyltransferase 1α.

## Competing interests

The authors declare that they have no competing interests.

## Authors’ contributions

DZ was responsible for all aspects of the project, including study design, experiments, statistical analysis, and manuscript preparation. FTH and GH were involved in the co-design of the work as well as the draft of the manuscript. YJZ carried out the cell studies. GZW and CJH participated in the molecular studies. YZ and MG carried out analytical work and contributed in drafting the manuscript. All authors read and approved the final manuscript.

## Authors’ information

DZ is a Ph.D. student; FTH, GH, YZ and MG are Ph.D. YJZ is a medical scientific researcher. GZW and CJH are Ph.D. students.

## Supplementary Material

Additional file 1: Figure S1 MiR-613 decreases TO901317-activated LXRα expression at both mRNA and protien levels in L02 cells. 12 hours after transfected with 80 nM miR-613 mimic or NC, L02 cells were treated with TO901317 (5 μM) for 24 hours. Real-time PCR analysis for LXRα mRNA level (A) and Western blot analysis for LXRα protein level (B). The relative level of LXRα expression determined using the 2-^△△CT^ method. *, *P* < 0.05 (n = 3 for each group). Click here for file

Additional file 2: Figure S2 MiR-613 suppresses LXRα-induced lipogenic genes in L02 cells. A, L02 cells were treated with TO901317 (5 μM) for 24 hours. Real-time PCR analysis for SREBP-1c, FAS, ChREBP and ACC mRNA level. B, after 12 hours transfected with 80 nM miR-613 mimic or NC, L02 cells were treated with TO901317 (5 μM) for 24 hours. Real-time PCR analysis for SREBP-1c, FAS, ChREBP and ACC mRNA level. The relative level of lipogenic gene expression determined using the 2-^△△CT^ method. *, *P* < 0.05 (n = 3 for each group). Click here for file
