# Supplementary figures and images for: MicroRNA-613 represses lipogenesis in HepG2 cells by downregulating LXRα
Source: Lipids Health Dis. 2013 Mar 8;12:32. doi: 10.1186/1476-511X-12-32 (PMC3605323; doi:10.1186/1476-511X-12-32)

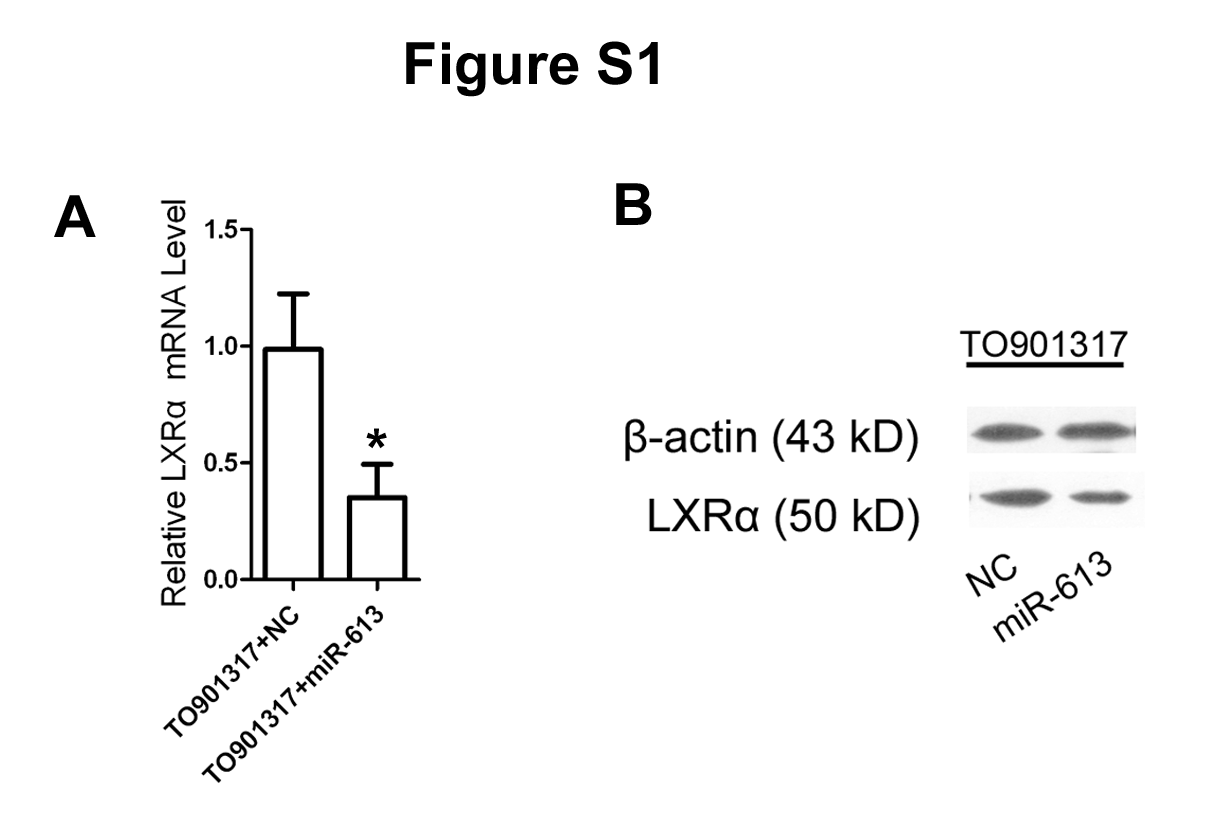

Supplement: Additional file 1: Figure S1 — MiR-613 decreases TO901317-activated LXRα expression at both mRNA and protien levels in L02 cells. 12 hours after transfected with 80 nM miR-613 mimic or NC, L02 cells were treated with TO901317 (5 μM) for 24 hours. Real-time PCR analysis for LXRα mRNA level (A) and Western blot analysis for LXRα protein level (B). The relative level of LXRα expression determined using the 2-△△CT method. *, P < 0.05 (n = 3 for each group). [file 1476-511X-12-32-S1.tiff]

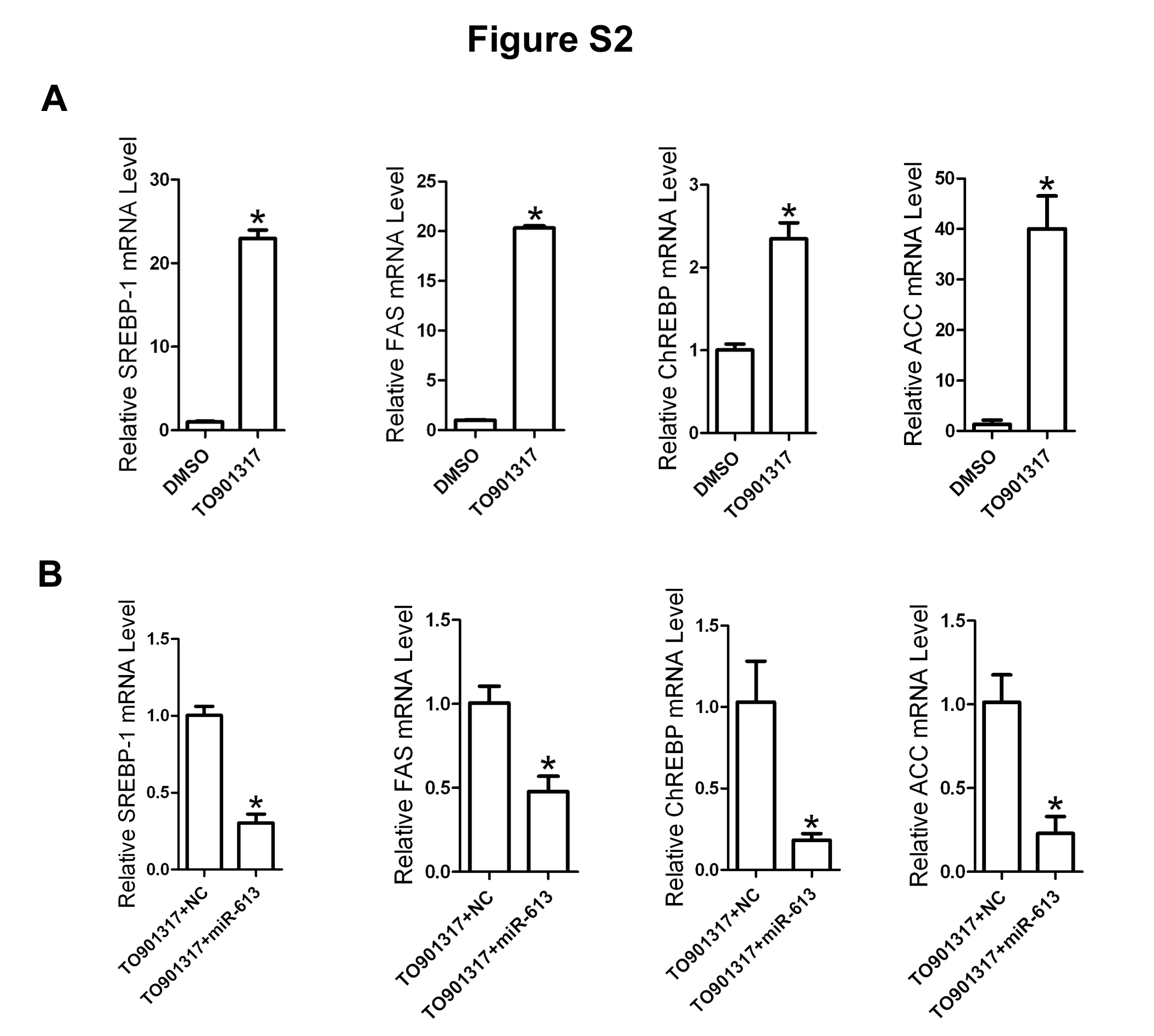

Supplement: Additional file 2: Figure S2 — MiR-613 suppresses LXRα-induced lipogenic genes in L02 cells. A, L02 cells were treated with TO901317 (5 μM) for 24 hours. Real-time PCR analysis for SREBP-1c, FAS, ChREBP and ACC mRNA level. B, after 12 hours transfected with 80 nM miR-613 mimic or NC, L02 cells were treated with TO901317 (5 μM) for 24 hours. Real-time PCR analysis for SREBP-1c, FAS, ChREBP and ACC mRNA level. The relative level of lipogenic gene expression determined using the 2-△△CT method. *, P < 0.05 (n = 3 for each group). [file 1476-511X-12-32-S2.tiff]
